# Timing of postmastectomy radiation therapy in two-stage expander/implant-based breast reconstruction: a systematic review and meta-analysis for the 2022 Japanese breast cancer society clinical practice guideline

**DOI:** 10.1007/s12282-025-01785-5

**Published:** 2025-09-25

**Authors:** Mami Ogita, Subaru Sawayanagi, Haruka Jinnouchi, Michio Yoshimura, Chikako Yamauchi, Naoko Sanuki, Yasushi Hamamoto, Kimiko Hirata, Mariko Kawamura, Yutaka Yamamoto, Shigehira Saji, Tatsuya Toyama

**Affiliations:** 1 Department of Radiology, The University of Tokyo Hospital, 7-3-1 Hongo, Bunkyo-Ku, Tokyo 113-8655 Japan; 2 Department of Radiology, Tokyo Teishin Hospital, 2-14-23 Fujimi, Chiyoda-Ku, Tokyo 102-8798 Japan; 3 Department of Radiation Oncology and Image-Applied Therapy, Graduate School of Medicine, Kyoto University, 54 Shogoin-Kawahara-Cho, Sakyo-Ku, Kyoto, Kyoto 606-8507 Japan; 4 Department of Radiation Oncology, Shiga General Hospital, 5-4-30 Moriyama, Moriyama, Shiga 524-8524 Japan; 5 Department of Radiology, Keio University School of Medicine, 35 Shinanomachi, Shinjuku-Ku, Tokyo 160-8582 Japan; 6 Department of Radiation Oncology, National Hospital Organization Shikoku Cancer Center, Ko-160, Minamiumemoto-Machi, Matsuyama, Ehime 791-0280 Japan; 7 Department of Radiation Therapy, Osaka Red Cross Hospital, 5-30 Fudegasakicho, Tennouji-Ku, Osaka, Osaka 543-8555 Japan; 8 Department of Radiology, Nagoya University Graduate School of Medicine, 65 Tsurumai-Cho, Showa-Ku, Nagoya, Aichi 466-8550 Japan; 9 Department of Breast and Endocrine Surgery, Graduate School of Medical Sciences, Kumamoto University, 1-1-1 Honjo, Chuo-Ku, Kumamoto, Kumamoto 860-8556 Japan; 10 Department of Medical Oncology, Fukushima Medical University, 1 Hikarigaoka, Fukushima, Fukushima 960-1295 Japan; 11 Department of Breast Surgery, Nagoya City University, 1 Kawasumi, Mizuho-Cho, Mizuho-Ku, Nagoya, Aichi 467-8601 Japan

**Keywords:** Breast cancer, Radiotherapy, Mammaplasty, Reconstruction, Tissue-expansion device, Implant

## Abstract

**Background:**

In immediate expander/implant reconstruction, postmastectomy radiation therapy (PMRT) can be administered at two different time points: during tissue expander insertion or after exchange with an implant. The optimal timing for PMRT remains unclear. We conducted a systematic review and meta-analysis to evaluate the impact of PMRT timing on the outcomes of patients with breast cancer undergoing two-stage expander/implant breast reconstruction.

**Methods:**

A literature review of articles in English and Japanese up to March 2021 was performed using PubMed/MEDLINE, the Cochrane Library, and Ichushi-Web. Studies comparing the timing of PMRT in patients with breast cancer undergoing immediate two-stage expander/implant breast reconstructions and PMRT were included. The assessed outcomes included major complications, reconstruction failure, cosmesis, capsular contractures, and local recurrence.

**Results:**

Eleven studies encompassing 1,628 cases were identified. These included one prospective cohort study, one prospective case–control study, and nine retrospective cohort studies. No significant differences were observed in major complications between PMRT to expander and PMRT to implant (odds ratio [OR] 1.11, 95% confidence intervals [CI] 0.72–1.73, *P* = 0.64). Reconstruction failure was more prevalent in the expander group (OR 2.33, 95% CI 1.43–3.82, *P* = 0.0007), while severe capsular contractures occurred less frequently in the expander group (OR 0.33, 95% CI 0.12–0.92, *P* = 0.03).

**Conclusions:**

PMRT to expander was associated with higher reconstruction failure and lower severe capsular contracture rates, with no significant differences in major complications by timing. There is insufficient evidence to favor one approach over the other.

**Supplementary Information:**

The online version contains supplementary material available at 10.1007/s12282-025-01785-5.

## Introduction

Breast reconstruction plays a pivotal role in the multidisciplinary treatment of breast cancer. Restoring breast appearance after mastectomy can alleviate the psychological burden associated with surgery and help maintain a patient’s positive body image and quality of life [1, 2]. The number of patients opting for breast reconstructions has increased in recent decades, particularly in favor of implant-based reconstruction [3]. Given the numerous advantages of implant-based breast reconstructions, including shorter surgical times, reduced hospital stays, expedited recovery periods, and absence of scars at the donor site [4], implant-based reconstruction has become increasingly preferred.

Postmastectomy radiation therapy (PMRT) has been established as the standard treatment for patients with node-positive breast cancer following mastectomy, as supported by several randomized controlled trials and meta-analyses demonstrating its efficacy in reducing breast cancer recurrence and mortality in patients with positive axillary lymph nodes [5–12]. Consequently, PMRT is recommended for patients who are likely to benefit from it, irrespective of the presence of breast reconstructions. However, there is a concern that PMRT may lead to an increased incidence of complications in the reconstructed breast.

Previous meta-analyses have shown that patients who receive PMRT for implant-based breast reconstruction experience more postoperative complications than those who do not [13–16]. In addition, growing evidence suggests that autologous reconstruction may provide better outcomes than implant-based reconstruction in the setting of PMRT [17]. Therefore, for patients who choose implant-based reconstruction, it is important to develop treatment strategies that minimize reconstruction-related complications without compromising the oncological benefit of PMRT.

Implant-based breast reconstruction is typically performed in one or two stages. In one-stage direct-to-implant reconstruction, a permanent implant is placed immediately during the mastectomy, whereas in two-stage reconstruction, a tissue expander is initially placed and subsequently replaced by a permanent implant [18]. Two-stage breast reconstruction techniques have become increasingly popular in recent years [19]. In immediate two-stage reconstruction, PMRT can be performed either during the expander phase or after exchange with the permanent implant. The timing of PMRT is influenced by various factors, including perioperative chemotherapy (neoadjuvant or adjuvant) and the duration of tissue expansion. Although previous systematic reviews have examined the impact of PMRT timing on two-stage implant-based breast reconstruction [20–22], their findings were inconsistent across studies and the optimal timing for PMRT remains controversial. Therefore, we conducted an updated systematic review and meta-analysis by incorporating recent studies, standardizing outcome definitions, and focusing on clinically relevant endpoints to evaluate the effect of PMRT timing on complications and clinical outcomes in breast cancer patients undergoing immediate two-stage breast reconstruction.

## Materials and methods

We report our findings based on the Preferred Reporting Items for Systematic Reviews and Meta-Analyses (PRISMA) guidelines [23]. This meta-analysis was conducted as part of the 2022 edition of the Japanese Breast Cancer Society Clinical Practice Guidelines for Radiation Treatment of Breast Cancer. This study was registered with PROSPERO under registration number CRD42021253388.

### Eligibility criteria

We included controlled trials and observational studies that assessed the effect of PMRT timing on immediate two-stage expander/implant breast reconstruction in breast cancer patients. Articles comparing two groups, one that received radiation to implants and the other to expanders, were included in the analysis. The exclusion criteria were review articles, case reports involving fewer than ten patients in one arm, single-arm studies, studies reporting preoperative radiotherapy, studies regarding delayed breast reconstruction and reconstruction after PMRT, studies focused on one-stage (direct-to-implant) breast reconstruction, and studies that did not evaluate outcomes compatible with this review. We limited our inclusion to studies published in English or Japanese.

### Search strategy

A systematic search was conducted using PubMed/MEDLINE, the Cochrane Library, and Ichushi-Web databases from January 2016 to March 2021. The final search was performed on 25 May 2021. A combination of MeSH terms and keywords, including "Breast Neoplasms," "Radiotherapy," "Mammaplasty," "Breast Implants," "Tissue Expansion Devices," and "Reconstruction," was employed in the literature search (Supplementary Table 1). Additionally, a manual search of the reference lists of relevant review articles was performed. The systematic review and meta-analysis were conducted by searching PubMed/MEDLINE, the Cochrane Library, and Ichushi-Web from the earliest records up to November 2016 using similar keywords to develop the Japanese Breast Cancer Society clinical practice guidelines for breast cancer published in 2018 [9]. This review was conducted to update previous systematic reviews and meta-analyses. The studies selected in the previous meta-analysis were reevaluated and included in this analysis if they met the established eligibility criteria.

### Selection and data collection process

During the primary screening phase, two independent reviewers (SS and HJ) reviewed the titles and abstracts of the identified studies and excluded those that did not meet the eligibility criteria. In the secondary screening phase, the remaining studies were reviewed by the same two reviewers (SS and HJ). Data were extracted by independent reviewers using a standardized form. Discrepancies between the reviewers were resolved through discussion, and if a consensus could not be achieved, a third reviewer (MO) intervened. In cases in which multiple articles were published on the same patient population, the article with the largest sample size was selected.

### Outcomes

The primary outcomes of this review were complications, cosmetic results (cosmesis), and local recurrence. Data on major complications, reconstruction failure, and capsular contractures were extracted. Major complications were defined as those requiring surgical interventions or hospitalization. Reconstruction failure was defined as the permanent removal of the expander or implant or conversion to autologous reconstruction. Capsular contractures were defined as a capsular contracture of Baker grade III or IV, or requiring additional surgery. We considered “good” and above as retained cosmesis, and less than “good” as a decline in cosmesis.

### Risk of bias and certainty assessment

Two independent reviewers (SS and HJ) used the Medical Information Distribution Service (MINDS) tool to assess the risk of bias in nonrandomized studies [24]. Each individual study was evaluated for indirectness, inconsistency, imprecision, and publication bias, contributing to an overall assessment of the risk of bias. A body of evidence was systematically established for each outcome.

### Data synthesis

This meta-analysis was conducted to compare events between PMRT to expanders (experimental group) and PMRT to implants (control group). Random effects models employing the inverse variance method were used to calculate odds ratios (OR) and corresponding 95% confidence intervals (CIs). Funnel plots were generated to assess the publication bias. All statistical tests were two-sided, and a P-value < 0.05 was considered statistically significant. Statistical analyses were performed using Review Manager (RevMan) v5.4 software by the Cochrane Collaboration.

## Results

### Study selection

**Fig. 1:**
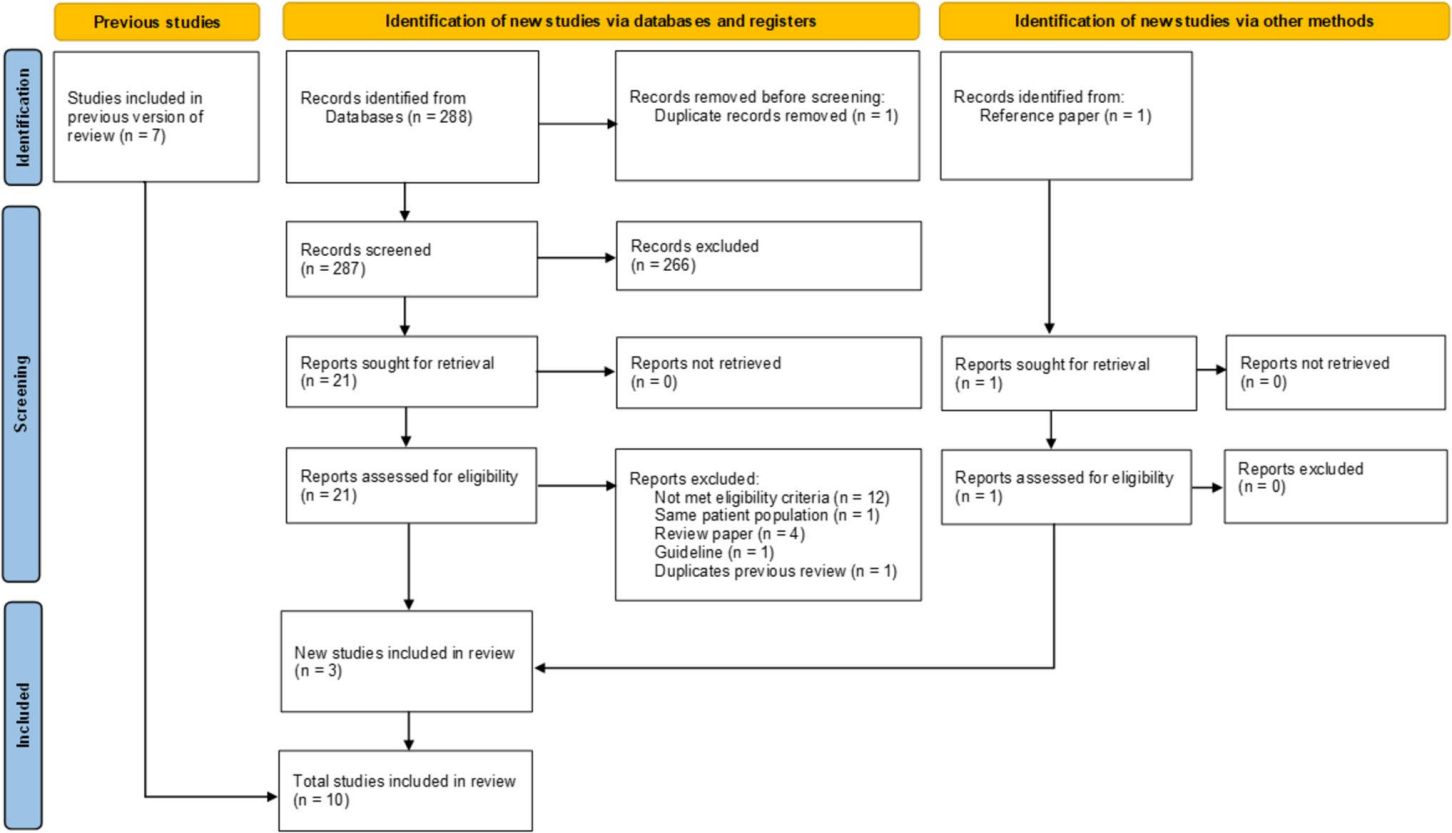
Flow diagram of literature screening according to PRISMA 2020 guidelines

The search conducted in PubMed/MEDLINE, the Cochrane Library, and Ichushi-Web yielded 288 citations. After duplicates were removed, 287 citations remained. A review of titles and abstracts led to the exclusion of 266 studies that did not meet the eligibility criteria. The full texts of 21 studies underwent detailed review. Nineteen studies were excluded for the following reasons: 12 studies did not satisfy the eligibility criteria, 5 studies were review articles or guidelines, one study involved the same patient population, and one study was a duplicate of a prior meta-analysis. Two studies that met the inclusion criteria and another identified by reviewing the reference papers were included. Consequently, a total of three studies from the new literature search and seven studies from previous analyses were included in this meta-analysis. The flow diagram is shown in Fig. 1.

### Study characteristics

This review included one case–control study [25], one prospective study [26], and eight retrospective cohort studies [27–34], published between 2009 and 2020. The characteristics of the studies are summarized in Table 1. The included studies were conducted in both single-center and multi-center settings. Chemotherapy was administered in most cohorts; however, the type and timing were generally unreported, and only four studies specified whether it was delivered in the adjuvant or neoadjuvant setting (Table 1). Three studies reported the time intervals between primary surgery (expander insertion), PMRT, and implant exchange. The detailed data are presented in Supplementary Table 2.

**Table 1:** Characteristics of the included studies

Study	Institution	Study period	Study design	Number of patients or breasts for analysis	Median follow-up period (months)	RT Dose to reconstructed breast (Gy)	Boost	Bolus	Chemotherapy	Endocrine therapy
Anderson, P.R. 2009	Fox chase cancer center, US	1987–2006	Retrospective cohort	Total 74 TE 62, PI 12	48 TE 36, PI 23	50–50.4	1%	100%	86%	59%
Nava, M.B. 2011	Fondazione IRCCS Istituto Nazionale Dei Tumori, Italy	2003–2007	Case–control	Total 159 TE 50, PI 109	50	50	NA	NA	100%	NA
Lentz, R. 2013	Yale New Haven Hospital, US	2004–2011	Retrospective cohort	Total 56 TE 34, PI 22	TE 27.3, PI 46.0*	NA	2%	NA	TE, NAC 50%, Adj 74%, PI, NAC 36%, Adj 77%	NA
Collier, P. 2014	St. John hospital and medical center, US	2006–2013	Retrospective cohort	Total 54 TE 32, PI 22	TE 16.3, PI 25.1*	NA	NA	NA	NA	NA
Cordeiro, P.G. 2015	Memorial Sloan Kettering Cancer Center, US	2003–2012	Retrospective cohort	Total 304 TE 94, PI 210	TE 30.1, PI 40.3*	NA	NA	Yes	100%	NA
Fowble, B. 2015	University of California, San Francisco, US	2004–2012	Retrospective cohort	Total 99 TE 86, PI 13	45.6	50–50.4	0%	Yes	98% NAC 52%, Adj 44%	78%
Yan, C. 2016	University of Pennsylvania, US	2005–2013	Retrospective cohort	Total 52 TE 41, PI 11	TE 21.8, PI 15.1	TE 52.6, PI 50.4*	17% TE 27%, PI 9%	17% TE15%, PI 27%	TE 93%, PI 98%	NA
Ogita, M. 2018	St. Luke’s International Hospital, Japan	2003–2014	Retrospective cohort	Total 81 TE 32, PI 49	32	50	4%	9%	Total 99% TE, NAC 88%, Adj 9%, PI, NAC 18%, Adj 84%	NA
Yuce Sari, S. 2019	Hacettepe University and Baskent University, Turkey	2004–2016	Retrospective cohort	Total 171 TE 17, PI 154	46.8	50	0.6%	71%	100% NAC 15%, Adj 85%	81%
Yoon, A.P. 2020	Multi-center, US	2012–2015	Prospective cohort	Total 317 TE 237, PI 80	At least 24	NA	NA	NA	TE 61%, PI 93% (during or after reconstruction)	NA

*RT* Radiation therapy, *TE* Tissue expander, *PI* Permanent implant, *NA* Not applicable, *NAC* Neoadjuvant chemotherapy, *Adj* Adjuvant chemotherapy

^*^Mean value

### Risk of bias assessment

The risk of bias, indirectness, and inconsistency for each outcome in the individual studies are presented in Supplementary Tables 3, 4, 5, and 6. Owing to the non-blinded and non-randomized nature of the observational studies and the absence of adjustment for confounding variables in all but one study, a risk of bias was determined to be present. The indirectness of all the outcomes was low. Inconsistency was low, except for capsular contractures, which exhibited moderate suspicion of inconsistency. Publication bias was noted, excluding reconstruction failure (Supplementary Figs. 1, 2, 3, and 4).

## Results of synthesis

### Major complications

**Fig. 2:**
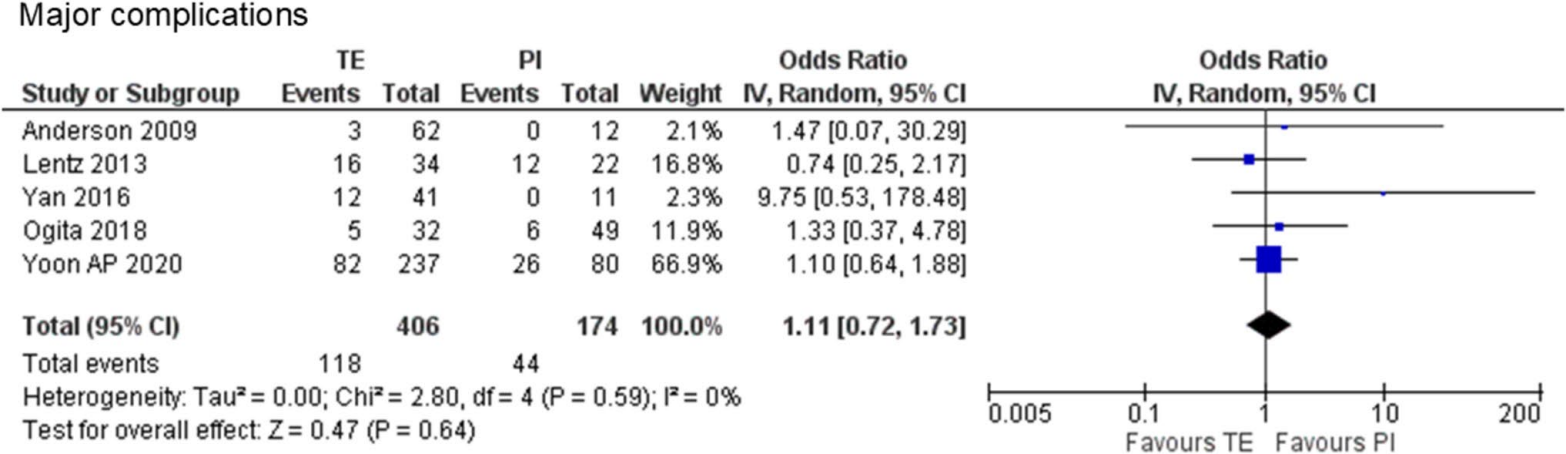
Forest plot illustrating the impact of postmastectomy radiation therapy timing on major complications requiring surgical intervention or hospitalization. *TE* Tissue expander, *PI* Permanent implant, *IV* Inverse variance

One prospective study [26] and four retrospective cohort studies [27, 28, 32, 33] encompassing 580 cases (406 cases of PMRT to expanders and 174 cases of PMRT to implants) were analyzed. No statistically significant difference was observed in major complications requiring surgical interventions or hospitalization between PMRT to expanders and PMRT to implants (29.1% vs. 25.3%, odds ratio [OR] 1.11, 95% confidence intervals [CI] 0.72–1.73, P = 0.64), with no detected heterogeneity (χ^2^ = 2.8, df = 4, P = 0.59, I^2^ = 0%) (Fig. 2).

### Reconstruction failure

**Fig. 3:**
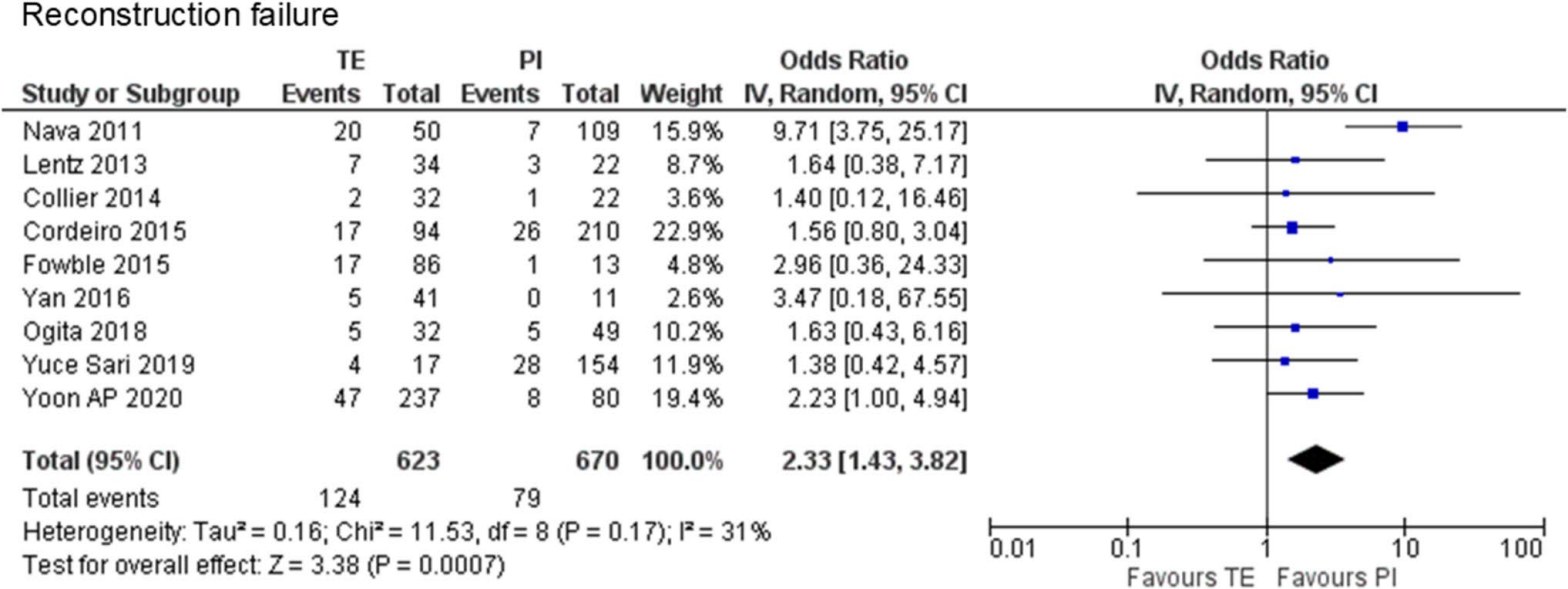
Forest plot illustrating the impact of postmastectomy radiation therapy timing on reconstruction failure. *TE* Tissue expander, *PI* Permanent implant, *IV* inverse variance

One case–control study [25], one prospective study [26], and seven retrospective cohort studies [28–34] were used, encompassing 1,293 cases (623 cases of PMRT to expanders and 670 cases of PMRT to implants) included in the analysis. The data indicated that PMRT to expanders was associated with a statistically significant higher rate of reconstruction failure compared to that with PMRT to implants (19.9% vs. 11.8%, OR 2.33, 95% CI 1.43–3.82, P = 0.0007), with no major heterogeneity observed (χ^2^ = 11.53, df = 8, P = 0.17, I^2^ = 31%) (Fig. 3).

### Capsular contractures

**Fig. 4:**
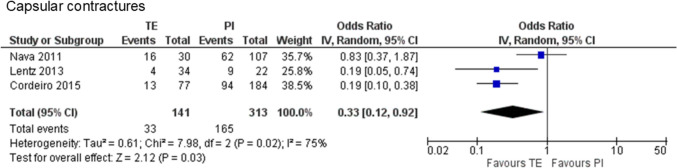
Forest plot illustrating the impact of postmastectomy radiation therapy timing on capsular contractures (Baker grade III or IV or requiring additional surgery). *TE* Tissue expander, *PI* Permanent implant, *IV* Inverse variance

One case–control study [25] and two retrospective cohort studies [28, 30] with 444 cases (141 cases of PMRT to expanders and 313 cases of PMRT to implants) were included in the analysis. The findings revealed that PMRT to expanders was associated with significantly less capsular contractures (Baker grade III or IV or requiring additional surgery) than did PMRT to implants (23.4% vs. 52.7%, OR 0.33, 95% CI 0.12–0.92, P = 0.03), with substantial heterogeneity present (χ^2^ = 7.98, df = 2, P = 0.02, I2 = 75%) (Fig. 4).

### Cosmesis

**Fig. 5:**
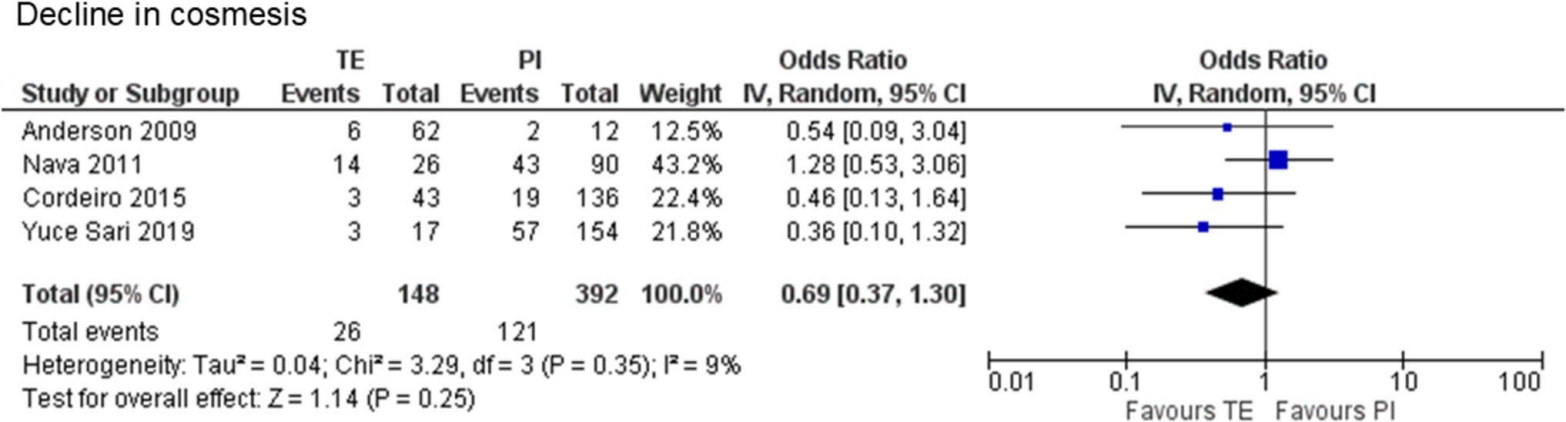
Forest plot illustrating the impact of postmastectomy radiation therapy timing on decline in cosmesis. *TE* Tissue expander, *PI* Permanent implant, *IV* Inverse variance

One case–control study [25] and three retrospective cohort studies [27, 30, 34] were integrated into the analysis, with 540 cases (148 cases of PMRT to expanders and 392 cases of PMRT to implants). Although no statistically significant difference in cosmesis with less than “good” was observed between PMRT to expanders and PMRT to implants (17.6% vs. 30.9%, OR 0.69, 95% CI 0.37–1.30, P = 0.25), no heterogeneity was found (χ^2^ = 3.29, df = 3, P = 0.35, I^2^ = 9%). However, there was a trend indicating a smaller decline in cosmesis for PMRT to the expanders (Fig. 5).

### Local recurrence

Only one retrospective cohort study reported breast cancer recurrence [33]. Among the 32 patients who received PMRT to expanders and 49 patients who received PMRT to implants, none experienced locoregional recurrence, with a median follow-up of 32 months. Seven patients (four in the PMRT to expander group and three in the PMRT to implant group) developed distant metastases. Owing to the limited number of available studies, a meta-analysis of local recurrence was not conducted.

### Certainty of evidence

Supplementary Table6 presents the overall evidence for each outcome. The certainty of evidence was rated low across all outcomes, primarily because of study limitations and potential publication bias.

## Discussion

This meta-analysis the impact of the timing of PMRT on outcomes in immediate two-stage expander/implant-based breast reconstruction. Several previous systematic reviews and meta-analyses have addressed the timing of PMRT in relation to implant-based reconstruction; however, their conclusions remain inconclusive because of the considerable diversity in outcome definitions.

Ricci et al. reported a significantly increased risk of reconstruction failure in patients receiving PMRT to expanders compared with that of those treated after implant exchange (20% vs. 13.4%, risk ratio [RR] 2.33, 95% CI 1.24–4.35, P = 0.0083), with non-significant but favorable trends toward reduced capsular contractures (24.5% vs. 49.4%, RR 0.53, 95% CI 0.26–1.09, P = 0.083) [20]. In contrast, Lee et al. showed a lower incidence of severe capsular contractures in the expander group (RR 0.44, 95% CI 0.29–0.68, P < 0.001), but found no statistically significant difference in reconstruction failure (RR 1.72, 95% CI 0.81–3.64, P = 0.16) and major complications (RR 3.29, 95% CI 0.76–4.25, P = 0.11) [21]. Guo et al. reported a significantly increased rate of reconstruction failure (RR 1.75, 95% CI, 1.03–2.98, P = 0.04) and a significantly decreased rate of capsular contractures (RR 0.43, 95% CI 0.32–0.58, P < 0.00001) in the expander group. Major complications were comparable between the groups (RR 1.19, 95% CI 0.83–1.70, P = 0.34) [22]. Variations in endpoint definitions, particularly regarding "reconstruction failure" and “major complications,” make it difficult to draw consistent clinical conclusions.

Therefore, we conducted an updated meta-analysis by integrating recent studies, applying consistent outcome criteria, and emphasizing clinically meaningful endpoints, including reconstruction failure, major complications, severe capsular contracture, cosmetic results, and oncological outcomes, which have not been evaluated in prior studies. Although Guo et al. published a more recent meta-analysis in 2022 that included overlapping studies, our study differs in methodology and scope. Guo et al. assessed study quality using the Newcastle–Ottawa Scale, whereas we used the MINDS tool for a systematic and GRADE-consistent risk-of-bias assessment and included additional clinically relevant endpoints that were not addressed in their analysis. Our findings showed that PMRT delivered to expanders was associated with a significantly higher risk of reconstruction failure and a reduced incidence of severe capsular contractures, whereas the rates of major complications did not differ between the groups.

PMRT to expanders has often been avoided due to concerns about a potential increase in reconstruction failure, leading to a preference for implant irradiation. This preference may sometimes delay PMRT until after expander–implant exchange, which could adversely affect oncologic outcomes. Our findings suggest that PMRT to implants may increase the risk of severe capsular contracture, and neither option is clearly superior. These results provide important evidence to support decision-making for patients and breast surgeons considering reconstruction, patient preferences, alternative treatment options, and the need to avoid unnecessary delays in PMRT. There are advantages and disadvantages associated with the timing of PMRT, and no single approach has been universally deemed superior. A consensus statement from an expert panel indicated that while the evidence related to the optimal sequencing between expander exchange and radiotherapy remains ambiguous, a lower failure rate is associated with PMRT administered to the implant, although this conclusion is based on low-level evidence, and they accepted the administration of PMRT to either the expander or implant [35].

The timing of irradiation is frequently influenced by perioperative chemotherapy. In patients undergoing neoadjuvant chemotherapy, PMRT is typically delivered to the expander immediately after surgery. In contrast, in an adjuvant chemotherapy setting, the expander is inflated during chemotherapy, and PMRT is delivered after replacement with an implant. In clinical practice, PMRT is occasionally delayed until after expander-to-implant exchange because of concerns about reconstruction failure associated with expander irradiation. However, such treatment delays should be avoided as they may compromise oncological outcomes.

Physical and dosimetric concerns are associated with the delivery of PMRT to expanders. Most tissue expanders contain a magnetic port used for saline injections, which may influence the dose distribution. The presence of dense materials can increase attenuation, scattering, and artifact generation on computed tomography images, potentially affecting the accuracy of treatment planning and dose calculations, particularly in areas adjacent to magnetic ports. The presence of the internal metallic port reduced the dose to the clinical target volume by an average of 11.7% [36]. These physical factors should be considered when evaluating the safety and efficacy of PMRT to expanders. However, new nonmetallic tissue expanders have recently become available, which may resolve concerns related to treatment planning.

This study had several limitations. Most studies included in the analysis were retrospective, and no randomized trials were identified. Consequently, the overall level of evidence was low, and there was a potential for selection bias. Most included studies carried a moderate to high risk of bias. In addition, data on the timing of PMRT in relation to surgery and chemotherapy were limited, which prevented further analysis of their potential associations with outcomes. Although statistical heterogeneity was low for most outcomes, substantial clinical heterogeneity may exist due to differences in study design, patient characteristics, surgical procedures, and adjuvant treatment regimens. A prospective randomized trial is warranted to ascertain the optimal timing; however, timing randomization is challenging in practice and high-quality evidence from randomized controlled trials is unlikely to emerge. We concluded that it was appropriate to integrate the available observational study evidence, but caution is needed when interpreting the results.

There are some risks of adverse events associated with PMRT to both expanders and implants. Consequently, patients must be adequately informed about the potential risks of PMRT for implant-based breast reconstructions and should receive treatment at an appropriate time that does not compromise oncological outcomes.

In conclusion, compared with PMRT delivered to implants, PMRT delivered to expanders was associated with an increased risk of reconstruction failure and a decreased risk of severe capsular contractures. No significant differences in major complications or cosmetic outcomes were observed between groups.

## Supplementary Information

Below is the link to the electronic supplementary material.

Supplementary file1 (DOCX 81 KB)

## Data Availability

All data used in this meta-analysis were extracted from publicly available articles cited in the manuscript. The studylevel extraction dataset compiled for this review is available from the corresponding author upon reasonable request.

## Declarations

### Research involving human and/or animals participants

This article does not contain any studies with human participants or animals performed by any of the authors.
