# Supplementary material for: Timing of postmastectomy radiation therapy in two-stage expander/implant-based breast reconstruction: a systematic review and meta-analysis for the 2022 Japanese breast cancer society clinical practice guideline
Source: Breast Cancer. 2025 Sep 25;32(6):1159–68. doi: 10.1007/s12282-025-01785-5 (PMC12552263; doi:10.1007/s12282-025-01785-5)
Supplement: Supplementary file 1 — Supplementary file1 (DOCX 81 KB) [file 12282_2025_1785_MOESM1_ESM.docx]

**Supplementary Appendix**

Supplementary Table 1. Search strategies on PubMed/MEDLINE and the Cochrane Library

| No | Search Strategy on PubMed/MEDLINE | Result |
| --- | --- | --- |
| #01 | "Breast Neoplasms/radiotherapy"[Mesh] OR ("Breast Neoplasms/therapy"[Mesh] AND "Radiotherapy"[Mesh]) | 20,211 |
| #02 | "Mammaplasty"[Mesh] | 14,682 |
| #03 | "Tissue Expansion Devices"[Mesh] OR "Breast Implants"[Mesh] | 6,654 |
| #04 | #1 AND #2 AND #3 | 272 |
| #05 | (Breast[TIAB] OR Mammary[TI]) AND (Tumo*[TIAB] OR Cancer*[TIAB] OR Carcinoma*[TIAB] OR Neoplasm*[TW]) AND (mammaplast*[TIAB] OR mammoplast*[TIAB] OR reconstruction[TIAB]) AND (Radiotherapy[TIAB] OR "radiation therapy"[TIAB] OR irradiation[TIAB]) AND ("Tissue Expansion Device"[TIAB] OR "Tissue Expansion Devices"[TW] OR implant*[TIAB]) | 682 |
| #06 | #4 OR #5 | 764 |
| #07 | #6 AND 2016/1:2021/3[DP] | 329 |
| #08 | #7 AND (JAPANESE[LA] OR ENGLISH[LA]) | 320 |
| #09 | #8 AND ("Meta-Analysis"[PT] OR "Meta-Analysis as Topic"[Mesh] OR "meta-analysis"[TIAB]) | 11 |
| #10 | #8 AND ("Cochrane Database Syst Rev"[TA] OR "Systematic Review"[PT] OR "Systematic Reviews as Topic"[Mesh] OR "systematic review"[TIAB]) | 12 |
| #11 | #8 AND ("Practice Guideline"[PT] OR "Practice Guidelines as Topic"[Mesh] OR "Consensus"[Mesh] OR "Consensus Development Conferences as Topic"[Mesh] OR "Consensus Development Conference"[PT] OR guideline*[TI] OR consensus[TI]) | 7 |
| #12 | #9 OR #10 OR #11 | 23 |
| #13 | #8 AND ("Randomized Controlled Trial"[PT] OR "Randomized Controlled Trials as Topic"[Mesh] OR (random*[TIAB] NOT medline[SB])) | 7 |
| #14 | #8 AND ("Clinical Trial"[PT] OR "Clinical Trials as Topic"[Mesh] OR "Observational Study"[PT] OR "Observational Studies as Topic"[Mesh] OR (("clinical trial"[TIAB] OR "case control"[TIAB] OR "case comparison"[TIAB]) NOT medline[SB])) | 24 |
| #15 | (#13 OR #14) NOT #12 | 26 |
| #16 | #8 AND ("Epidemiologic Methods"[Mesh] OR "Comparative Study"[PT] OR "Multicenter Study"[PT] OR "Validation Study"[PT] OR ((cohort*[TIAB] OR "comparative study"[TIAB] OR "follow-up"[TIAB] OR "prospective study"[TIAB] OR "Retrospective study"[TIAB]) NOT medline[SB])) | 222 |
| #17 | #16 NOT (#12 OR #15) | 190 |

| No. | Search Strategy on the Cochrane Library | Result |
| --- | --- | --- |
| #1 | (Breast:ti OR Mammary:ti) AND (Tumo*:ti,ab,kw OR Cancer*:ti,ab,kw OR Carcinoma*:ti,ab,kw OR Neoplasm*:ti,ab,kw OR oncology:ti,ab,kw) | 29,262 |
| #2 | Radiotherapy:ti OR radiation:ti OR irradiation:ti | 20,971 |
| #3 | (mammaplast*:ti,ab,kw OR mammoplast*:ti,ab,kw OR reconstruction:ti,ab,kw) AND ("Tissue Expansion Device":ti,ab,kw OR "Tissue Expansion Devices":ti,ab,kw OR implant*:ti,ab,kw) | 885 |
| #4 | #1 AND #2 AND #3 | 11 |
| #5 | #3 publication date from Jan 2016 to Mar 2021 | 9 |
| #6 | #4 CDSR | 0 |
| #7 | #4 CCRCT | 9 |

Supplementary Table 2. Time intervals between primary surgery (tissue expander insertion), postmastectomy radiation therapy, and permanent implant exchange in the included studies

| Study | Interval from TE insertion* to PMRT (days) | Interval from PMRT to PI exchange (days) | Interval from PI exchange to PMRT (days) | Interval from TE insertion* to PI exchange (days) |
| --- | --- | --- | --- | --- |
| Lentz, R. 2013 | TE group: 188  PI group: 220 |  |  |  |
| Collier, P. 2014 | TE group: 113  PI group: 213 |  |  | TE group: 251  PI group: 165 |
| Ogita, M. 2018 | TE group: 51  PI group: NA | TE group: 248 | PI group: 29 |  |

*TE insertion was performed at the time of primary surgery.

TE: tissue expander; PMRT: postmastectomy radiation therapy; PI: permanent implant; NA: not applicable

TE group represents irradiation to TE. PI group represents irradiation to PI.

Supplementary Table 3. Risk of bias assessment for major complications

| Outcomes | | Major complications requiring a surgical intervention and/or hospitalization | | | | | |  |  |  |  |  |  |  | |  | |  | |  |  |  |  |  |  |  |  |  |  |
| --- | --- | --- | --- | --- | --- | --- | --- | --- | --- | --- | --- | --- | --- | --- | --- | --- | --- | --- | --- | --- | --- | --- | --- | --- | --- | --- | --- | --- | --- |
| Individual study | | Risk of bias | | | | | |  |  |  |  |  |  |  | |  | |  | |  |  |  |  |  |  |  |  |  |  |
|  |  | Selection bias | Performance bias | Detection bias | Attrition bias | Others | |  | Factors that can increase the certainty of the evidence | | |  | Indirectness | | | | | | |  | Number at risk (outcome rate) | | | | | |  |  |  |
| Study code | Study design | Differences between baseline characteristics | Differences between groups in the care | Differences between groups in how outcomes are determined | Differences between groups in withdrawals | Inadequate adjustment for confounding | Others | Summary | Dose-response gradient | Effect of potential residual confounding factors | Large magnitude of effect | Summary | Participants | | Intervention | | Comparison | | Outcome | Summary | The denominator for comparison arm | The numerator for comparison arm | (%) | The denominator for intervention arm | The numerator for intervention arm | (%) | Effect measures | An estimate of effect | Confidence interval |
| Anderson, PR. 2009 | Retrospective cohort | 0 | 0 | -2 | 0 | -2 | 0 | -1 | 0 | 0 | 0 | 0 | 0 | | 0 | | 0 | | 0 | 0 | 12 | 0 | 0 | 62 | 3 | 4.8 | OR | 1.47 | 0.07–30.29 |
| Lentz, R. 2013 | Retrospective cohort | -2 | 0 | -2 | 0 | -2 | 0 | -1 | 0 | 0 | 0 | 0 | 0 | | 0 | | 0 | | 0 | 0 | 22 | 12 | 54.5 | 34 | 16 | 47.1 | OR | 0.74 | 0.25–2.17 |
| Yan, C. 2016 | Retrospective cohort | -1 | 0 | -2 | 0 | -2 | 0 | -1 | 0 | 0 | 0 | 0 | 0 | | 0 | | 0 | | 0 | 0 | 11 | 0 | 0 | 41 | 12 | 29.3 | OR | 9.75 | 0.53–178.48 |
| Ogita, M. 2018 | Retrospective cohort | -2 | 0 | -2 | 0 | -2 | 0 | -1 | 0 | 0 | 0 | 0 | 0 | | 0 | | 0 | | 0 | 0 | 49 | 6 | 12.2 | 32 | 5 | 15.6 | OR | 1.33 | 0.37–4.78 |
| Yoon, AP. 2020 | Multicenter prospective | 0 | -1 | -2 | 0 | -2 | 0 | -1 | 0 | 0 | 0 | 0 | 0 | | 0 | | 0 | | 0 | 0 | 80 | 26 | 32.5 | 237 | 82 | 34.6 | OR | 1.1 | 0.64–1.88 |
| Comment |  | | | | | | |  |  |  |  |  |  | |  | |  | |  |  |  |  |  |  |  |  |  |  |  |
| Anderson, PR. 2009 |  |  |  | Unblinded |  | No adjustment for confounding was performed. |  |  |  |  |  |  |  | |  | |  | |  |  |  |  |  |  |  |  |  |  |  |
| Lentz, R. 2013 |  | Significant difference in the follow-up period. |  | Unblinded |  | No adjustment for confounding was performed. |  |  |  |  |  |  |  | |  | |  | |  |  |  |  |  |  |  |  |  |  |  |
| Yan, C. 2016 |  | TNM status unknown. |  | Unblinded |  | No adjustment was performed in the multivariate analysis. |  |  |  |  |  |  |  | |  | |  | |  |  |  |  |  |  |  |  |  |  |  |
| Ogita, M. 2018 |  | Significant difference in cStage. |  | Unblinded |  | No adjustment for confounding was performed. |  |  |  |  |  |  |  | |  | |  | |  |  |  |  |  |  |  |  |  |  |  |
| Yoon, AP. 2020 |  |  | The details of chemotherapy were not specified. | Unblinded |  | No TNM adjustment was performed in the multivariate analysis. |  |  |  |  |  |  |  | |  | |  | |  |  |  |  |  |  |  |  |  |  |  |

Supplementary Table 4. Risk of bias assessment for reconstruction failure

| Outcomes | | Reconstruction failure | | | | | |  |  |  |  |  |  |  |  |  |  |  |  |  |  |  |  |  |  |  |
| --- | --- | --- | --- | --- | --- | --- | --- | --- | --- | --- | --- | --- | --- | --- | --- | --- | --- | --- | --- | --- | --- | --- | --- | --- | --- | --- |
| Individual study | | Risk of bias | | | | | |  |  |  |  |  |  |  |  |  |  |  |  |  |  |  |  |  |  |  |
|  |  | Selection bias | Performance bias | Detection bias | Attrition bias | Others | |  | Factors that can increase the certainty of the evidence | | |  | Indirectness | | | |  | Number at risk (outcome rate) | | | | | |  |  |  |
| Study code | Study design | Differences between baseline characteristics | Differences between groups in the care | Differences between groups in how outcomes are determined | Differences between groups in withdrawals | Inadequate adjustment for confounding | Others | Summary | Dose-response gradient | Effect of potential residual confounding factors | Large magnitude of effect | Summary | Participants | Intervention | Comparison | Outcome | Summary | The denominator for comparison arm | The numerator for comparison arm | (%) | The denominator for intervention arm | The numerator for intervention arm | (%) | Effect measures | An estimate of effect | Confidence interval |
| Nava, MB. 2011 | Case-control study | 0 | -1 | -2 | 0 | -1 | 0 | -1 | 0 | 0 | 0 | 0 | 0 | 0 | 0 | 0 | 0 | 109 | 7 | 6.4 | 50 | 20 | 40.0 | OR | 9.71 | 3.75–25.17 |
| Lentz, R. 2013 | Retrospective cohort | -2 | 0 | -2 | 0 | -2 | 0 | -1 | 0 | 0 | 0 | 0 | 0 | 0 | 0 | 0 | 0 | 22 | 3 | 13.6 | 34 | 7 | 20.6 | OR | 1.64 | 0.38–7.17 |
| Collier, P. 2014 | Retrospective cohort | -1 | -1 | -2 | 0 | -2 | 0 | -1 | 0 | 0 | 0 | 0 | 0 | 0 | 0 | 0 | 0 | 22 | 1 | 4.5 | 32 | 2 | 6.3 | OR | 1.4 | 0.12–16.46 |
| Cordeiro, PG. 2015 | Retrospective cohort | -1 | -1 | -2 | 0 | -2 | 0 | -1 | 0 | 0 | 0 | 0 | 0 | 0 | 0 | 0 | 0 | 210 | 26 | 12.4 | 94 | 17 | 18.1 | OR | 1.56 | 0.80–3.04 |
| Fowble, B. 2015 | Retrospective cohort | -1 | -1 | -2 | 0 | -2 | 0 | -1 | 0 | 0 | 0 | 0 | 0 | 0 | 0 | 0 | 0 | 13 | 1 | 7.7 | 86 | 17 | 19.8 | OR | 2.96 | 0.36–24.33 |
| Yan, C. 2016 | Retrospective cohort | -1 | 0 | -2 | 0 | -2 | 0 | -1 | 0 | 0 | 0 | 0 | 0 | 0 | 0 | 0 | 0 | 11 | 0 | 0.0 | 41 | 5 | 12.2 | OR | 3.47 | 0.18–67.55 |
| Ogita, M. 2018 | Retrospective cohort | -2 | 0 | -2 | 0 | -2 | 0 | -1 | 0 | 0 | 0 | 0 | 0 | 0 | 0 | 0 | 0 | 49 | 5 | 10.2 | 32 | 5 | 15.6 | OR | 1.63 | 0.43–6.16 |
| Yuce Sari S, 2019 | Retrospective cohort | -1 | -1 | -2 | 0 | -2 | 0 | -1 | 0 | 0 | 0 | 0 | 0 | -1 | -1 | 0 | -1 | 154 | 28 | 18.2 | 17 | 4 | 23.5 | OR | 1.38 | 0.42–4.57 |
| Yoon, AP. 2020 | Multicenter prospective | 0 | -1 | -2 | 0 | -2 | 0 | -1 | 0 | 0 | 0 | 0 | 0 | 0 | 0 | 0 | 0 | 80 | 8 | 10.0 | 237 | 47 | 19.8 | OR | 2.23 | 1.00–4.94 |
| Comment | | | | | | |  |  |  |  |  |  |  |  |  |  |  |  |  |  |  |  |  |  |  |  |
| Nava, MB. 2011 |  |  | Not specified. | Unblinded |  | Some confounding factors were adjusted. |  |  |  |  |  |  |  |  |  |  |  |  |  |  |  |  |  |  |  |  |
| Lentz, R. 2013 |  | Significant difference in the follow-up period. |  | Unblinded |  | No adjustment for confounding was performed. |  |  |  |  |  |  |  |  |  |  |  |  |  |  |  |  |  |  |  |  |
| Collier, P. 2014 |  | Not specified, except for age. | Not specified. | Unblinded |  | No adjustment for confounding was performed. |  |  |  |  |  |  |  |  |  |  |  |  |  |  |  |  |  |  |  |  |
| Cordeiro, PG. 2015 |  | TNM status unknown. | The details of chemotherapy were not specified. | Unblinded |  | No adjustment was performed in the multivariate analysis. |  |  |  |  |  |  |  |  |  |  |  |  |  |  |  |  |  |  |  |  |
| Fowble, B. 2015 |  | Not specified. | Not specified. | Unblinded |  | No adjustment for confounding was performed. |  |  |  |  |  |  |  |  |  |  |  |  |  |  |  |  |  |  |  |  |
| Yan, C. 2016 |  | TNM status unknown. |  | Unblinded |  | No adjustment was performed in the multivariate analysis. |  |  |  |  |  |  |  |  |  |  |  |  |  |  |  |  |  |  |  |  |
| Ogita, M. 2018 |  | Significant difference in cStage. |  | Unblinded |  | No adjustment for confounding was performed. |  |  |  |  |  |  |  |  |  |  |  |  |  |  |  |  |  |  |  |  |
| Yuce Sari S, 2019 |  | TNM status unknown. | The details of chemotherapy were not specified. | Unblinded |  | No adjustment was performed in the multivariate analysis. |  |  |  |  |  |  |  | Includes chest wall only and 2D irradiation. | Includes chest wall only and 2D irradiation. |  |  |  |  |  |  |  |  |  |  |  |
| Yoon, AP. 2020 |  |  | The details of chemotherapy were not specified. | Unblinded |  | No TNM adjustment was performed in the multivariate analysis. |  |  |  |  |  |  |  |  |  |  |  |  |  |  |  |  |  |  |  |  |

Supplementary Table 5. Risk of bias assessment for capsular contracture

| Outcomes | | Capsular contracture with Baker grade III or IV and/or requiring additional surgery | | | | | |  |  |  | |  | |  |  |  | |  | |  | |  |  |  | |  | |  | |  | |  | |  |  |  |
| --- | --- | --- | --- | --- | --- | --- | --- | --- | --- | --- | --- | --- | --- | --- | --- | --- | --- | --- | --- | --- | --- | --- | --- | --- | --- | --- | --- | --- | --- | --- | --- | --- | --- | --- | --- | --- |
| Individual study | | Risk of bias | | | | | |  |  |  | |  | |  |  |  | |  | |  | |  |  |  | |  | |  | |  | |  | |  |  |  |
|  |  | Selection bias | Performance bias | Detection bias | Attrition bias | Others | |  | Factors that can increase the certainty of the evidence | | | | |  | Indirectness | | | | | | |  | Number at risk (outcome rate) | | | | | | | | | | |  |  |  |
| Study code | Study design | Differences between baseline characteristics | Differences between groups in the care | Differences between groups in how outcomes are determined | Differences between groups in withdrawals | Inadequate adjustment for confounding | Others | Summary | Dose-response gradient | | Effect of potential residual confounding factors | | Large magnitude of effect | Summary | Participants | | Intervention | | Comparison | | Outcome | Summary | The denominator for comparison arm | | The numerator for comparison arm | | (%) | | The denominator for intervention arm | | The numerator for intervention arm | | (%) | Effect measures | An estimate of effect | Confidence interval |
| Cordeiro, PG. 2015 | Retrospective cohort | -1 | -1 | -2 | 0 | -2 | 0 | -1 | 0 | | 0 | | 0 | 0 | 0 | | 0 | | 0 | | 0 | 0 | 184 | | 94 | | 51.1 | | 77 | | 13 | | 16.9 | OR | 0.19 | 0.10–0.38 |
| Lentz, R. 2013 | Retrospective cohort | -2 | 0 | -2 | 0 | -2 | 0 | -1 | 0 | | 0 | | 0 | 0 | 0 | | 0 | | 0 | | 0 | 0 | 22 | | 9 | | 40.9 | | 34 | | 4 | | 11.8 | OR | 0.19 | 0.05–0.74 |
| Nava, MB. 2011 | Case-control | 0 | -1 | -2 | 0 | -1 | 0 | -1 | 0 | | 0 | | 0 | 0 | 0 | | 0 | | 0 | | 0 | 0 | 107 | | 62 | | 57.9 | | 30 | | 16 | | 53.3 | OR | 0.83 | 0.37–1.87 |
| Comment | | | | | | |  |  |  | |  | |  |  |  | |  | |  | |  |  |  | |  | |  | |  | |  | |  |  |  |  |
| Cordeiro, PG. 2015 |  | TNM status unknown. | The details of chemotherapy were not specified. | Unblinded |  | No adjustment was performed in the multivariate analysis. |  |  |  | |  | |  |  |  | |  | |  | |  |  |  | |  | |  | |  | |  | |  |  |  |  |
| Lentz, R. 2013 |  | Significant difference in the follow-up period. |  | Unblinded |  | No adjustment for confounding was performed. |  |  |  | |  | |  |  |  | |  | |  | |  |  |  | |  | |  | |  | |  | |  |  |  |  |
| Nava, MB. 2011 |  |  | Not specified. | Unblinded |  | Some confounding factors were adjusted. |  |  |  | |  | |  |  |  | |  | |  | |  |  |  | |  | |  | |  | |  | |  |  |  |  |

Supplementary Table 6. Risk of bias assessment for cosmesis

| Outcomes | | Decline in cosmesis (less than "good") | | | | | |  |  |  |  |  |  |  |  |  |  |  |  |  |  |  |  |  |  |  |
| --- | --- | --- | --- | --- | --- | --- | --- | --- | --- | --- | --- | --- | --- | --- | --- | --- | --- | --- | --- | --- | --- | --- | --- | --- | --- | --- |
| Individual study | | Risk of bias | | | | | |  |  |  |  |  |  |  |  |  |  |  |  |  |  |  |  |  |  |  |
|  |  | Selection bias | Performance bias | Detection bias | Attrition bias | Others | |  | Factors that can increase the certainty of the evidence | | |  | Indirectness | | | |  | Number at risk (outcome rate) | | | | | |  |  |  |
| Study code | Study design | Differences between baseline characteristics | Differences between groups in the care | Differences between groups in how outcomes are determined | Differences between groups in withdrawals | Inadequate adjustment for confounding | Others | Summary | Dose-response gradient | Effect of potential residual confounding factors | Large magnitude of effect | Summary | Participants | Intervention | Comparison | Outcome | Summary | The denominator for comparison arm | The numerator for comparison arm | (%) | The denominator for intervention arm | The numerator for intervention arm | (%) | Effect measures | An estimate of effect | Confidence interval |
| Yuce Sari, S. 2019 | Retrospective cohort | -1 | -1 | -2 | 0 | -2 | 0 | -1 | 0 | 0 | 0 | 0 | 0 | -1 | -1 | 0 | -1 | 154 | 57 | 37.0 | 17 | 3 | 17.7 | OR | 0.36 | 0.10–1.32 |
| Cordeiro, PG. 2015 | Retrospective cohort | -1 | -1 | -2 | 0 | -2 | 0 | -1 | 0 | 0 | 0 | 0 | 0 | 0 | 0 | 0 | 0 | 136 | 19 | 14.0 | 43 | 3 | 7.0 | OR | 0.46 | 0.13–1.64 |
| Nava, MB. 2011 | Case-control | 0 | -1 | -2 | 0 | -1 | 0 | -1 | 0 | 0 | 0 | 0 | 0 | 0 | 0 | 0 | 0 | 90 | 43 | 47.8 | 26 | 14 | 53.9 | OR | 1.28 | 0.53–3.06 |
| Anderson, PR. 2009 | Retrospective cohort | 0 | 0 | -2 | 0 | -2 | 0 | -1 | 0 | 0 | 0 | 0 | 0 | 0 | 0 | 0 | 0 | 12 | 2 | 16.7 | 62 | 6 | 9.7 | OR | 0.54 | 0.09–3.04 |
| Comment |  | | | | | | |  |  |  |  |  |  |  |  |  |  |  |  |  |  |  |  |  |  |  |
| Yuce Sari, S. 2019 |  | TNM status unknown. | The details of chemotherapy were not specified. | Unblinded |  | No adjustment was performed in the multivariate analysis. |  |  |  |  |  |  |  | Includes chest wall only and 2D irradiation. | Includes chest wall only and 2D irradiation. |  |  |  |  |  |  |  |  |  |  |  |
| Cordeiro, PG. 2015 |  | TNM status unknown. | The details of chemotherapy were not specified. | Unblinded |  | No adjustment was performed in the multivariate analysis. |  |  |  |  |  |  |  |  |  |  |  |  |  |  |  |  |  |  |  |  |
| Nava, MB. 2011 |  |  | Not specified. | Unblinded |  | Some confounding factors were adjusted. |  |  |  |  |  |  |  |  |  |  |  |  |  |  |  |  |  |  |  |  |
| Anderson, PR. 2009 |  |  |  | Unblinded |  | No adjustment for confounding was performed. |  |  |  |  |  |  |  |  |  |  |  |  |  |  |  |  |  |  |  |  |

Supplementary Table 6. Body of evidence by each outcome

|  |  |  | |  | |  |  | |  | |  | | Number at risk (outcome rate) | | | | | |  |  |  |  |  |
| --- | --- | --- | --- | --- | --- | --- | --- | --- | --- | --- | --- | --- | --- | --- | --- | --- | --- | --- | --- | --- | --- | --- | --- |
| Outcomes | Study design /Number of studies | Risk of bias | Inconsistency | | Imprecision | | | Indirectness | | Others (Publication bias) | | Factors that can increase the certainty of the evidence | The denominator for comparison arm | The numerator for comparison arm | (％) | The denominator for intervention arm | The numerator for intervention arm | (％) | Effect measures | An estimate of effect | 95% Confidence interval | Certainty of evidence | Importance |
| Major complications | Cohort /5 | -1 | 0 | | -1 | | | 0 | | -1 | | 0 | 174 | 44 | 25.3 | 406 | 118 | 29.1 | OR | 1.11 | 0.72–1.73 | Low (C) | 8 |
| Reconstruction failure | Cohort /8,  Case-control /1 | -1 | 0 | | 0 | | | 0 | | 0 | | 0 | 670 | 79 | 11.8 | 623 | 124 | 19.9 | OR | 2.33 | 1.43–3.82 | Low (C) | 8 |
| Capsular contracture | Cohort /2,  Case-control /1 | -1 | -1 | | 0 | | | 0 | | -1 | | 0 | 313 | 165 | 52.7 | 141 | 33 | 23.4 | OR | 0.33 | 0.12–0.92 | Low (C) | 8 |
| Decline in cosmesis | Cohort /3,  Case-control /1 | -1 | 0 | | -1 | | | 0 | | -1 | | 0 | 392 | 121 | 30.9 | 148 | 26 | 17.6 | OR | 0.69 | 0.37–1.30 | Low (C) | 7 |


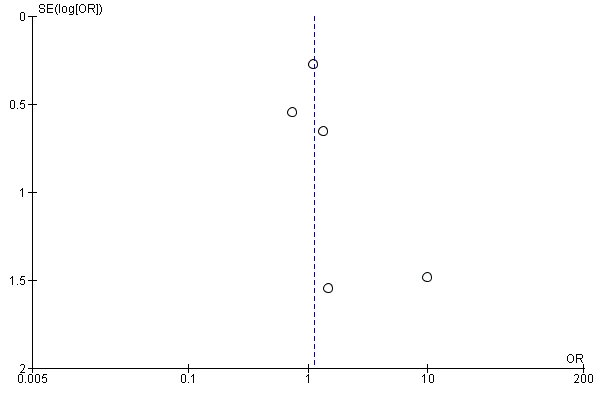


Supplementary Figure 1. Funnel plot evaluating publication bias for major complications


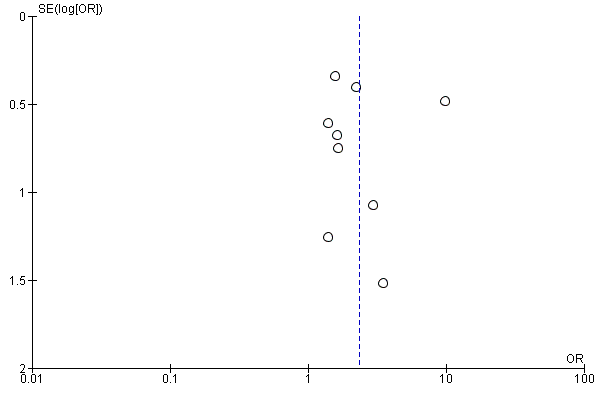


Supplementary Figure 2. Funnel plot evaluating publication bias for reconstruction failure


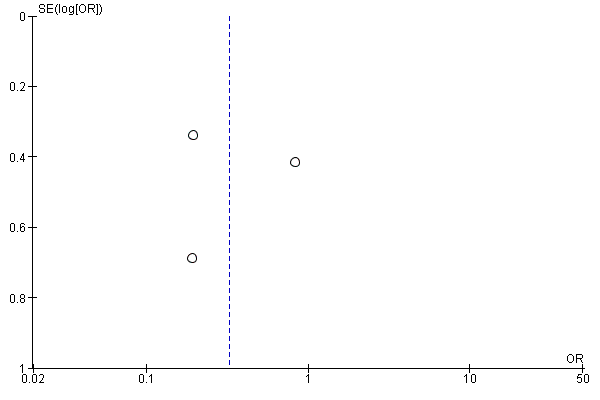


Supplementary Figure 3. Funnel plot evaluating publication bias for capsular contracture


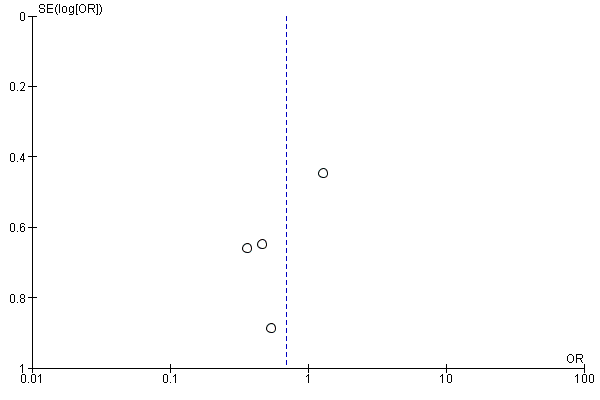


Supplementary Figure 4. Funnel plot evaluating publication bias for cosmesis
